# Leptin Signaling Affects Survival and Chemoresistance of Estrogen Receptor Negative Breast Cancer

**DOI:** 10.3390/ijms21113794

**Published:** 2020-05-27

**Authors:** Crystal C. Lipsey, Adriana Harbuzariu, Robert W. Robey, Lyn M. Huff, Michael M. Gottesman, Ruben R. Gonzalez-Perez

**Affiliations:** 1 Microbiology, Biochemistry, and Immunology, GEBS, Morehouse School of Medicine, Atlanta, GA 30310, USA; clipsey@msm.edu (C.C.L.); aharbuzariu@msm.com (A.H.); 2 Laboratory of Cell Biology, Center for Cancer Research, NCI, National Institutes of Health, Bethesda, MD 20892, USA; robeyr@nih.gov (R.W.R.); lyn@nih.gov (L.M.H.); gottesmm@nih.gov (M.M.G.)

**Keywords:** estrogen receptor negative breast cancer survival, leptin, leptin antagonist, obesity-related cancer, chemoresistance

## Abstract

Estrogen-receptor-negative breast cancer (BCER−) is mainly treated with chemotherapeutics. Leptin signaling can influence BCER− progression, but its effects on patient survival and chemoresistance are not well understood. We hypothesize that leptin signaling decreases the survival of BCER− patients by, in part, inducing the expression of chemoresistance-related genes. The correlation of expression of leptin receptor (OBR), leptin-targeted genes (CDK8, NANOG, and RBP-Jk), and breast cancer (BC) patient survival was determined from The Cancer Genome Atlas (TCGA) mRNA data. Leptin-induced expression of proliferation and chemoresistance-related molecules was investigated in triple-negative BC (TNBC) cells that respond differently to chemotherapeutics. Leptin-induced gene expression in TNBC was analyzed by RNA-Seq. The specificity of leptin effects was assessed using OBR inhibitors (shRNA and peptides). The results show that OBR and leptin-targeted gene expression are associated with lower survival of BCER− patients. Importantly, the co-expression of these genes was also associated with chemotherapy failure. Leptin signaling increased the expression of tumorigenesis and chemoresistance-related genes (ABCB1, WNT4, ADHFE1, TBC1D3, LL22NC03, RDH5, and ITGB3) and impaired chemotherapeutic effects in TNBC cells. OBR inhibition re-sensitized TNBC to chemotherapeutics. In conclusion, the co-expression of OBR and leptin-targeted genes may be used as a predictor of survival and drug resistance of BCER− patients. Targeting OBR signaling could improve chemotherapeutic efficacy.

## 1. Introduction

Obesity is a worldwide problem that increases the incidence and severity of many different medical conditions. The number of overweight adults worldwide is over 1.9 billion, with 650 million of those individuals classified as obese [1]. In 2018, the American Association for Cancer Research (AACR) reported that 40% of the cancer cases in the U.S. were classified as obesity-related cancers [2]. Currently, 13 different obesity-related cancers have been identified, including postmenopausal breast cancer (BC) [3].

Several aberrant mechanisms are believed to be involved in the development of obesity-related cancers. Among these mechanisms, the link between major proliferative pathways and excess amounts of adipose tissue has been investigated via the increased production and circulation of leptin, a 16 kDa protein adipokine [4,5,6]. Leptin is primarily secreted by adipocytes; thus, circulating leptin levels are increased in proportion to body fat. Sustained high levels of leptin can lead to a “leptin-resistance state” characterized by uncontrolled appetite and energy balance [7]. The leptin receptor, OBR, is found in hypothalamic cells, and it is expressed at very low levels in other normal cells. In contrast, several cancer cell types overexpress OBR, including BC and pancreatic cancer (PC) cells [5,8]. Leptin binding to OBR induces the recruitment of JAK2 kinase, which in turn activates several canonical signaling responses, including the phosphorylation of STAT3 [9]. pSTAT3 translocates to the nucleus and binds downstream genes [9] that regulate multiple signaling pathways essential for angiogenesis, proliferation, migration, and cell survival [10]. Leptin signaling upregulates Notch and its gatekeeper, the transcription factor RBP-Jk [11], and several pluripotent molecules that can induce cancer stem cell maintenance. Cancer stem cells are believed to contribute to chemoresistance and tumor relapse [10,12,13].

The development of dysfunctional adipose tissues in obese and overweight patients has been correlated with a lower risk of estrogen-receptor-positive BC (BCER+) but with a higher risk of estrogen-negative BC (BCER−) before menopause [14]. Estrogen binding to estrogen receptor (ER) initiates genomic activities and non-genomic effects that regulate downstream protein expression involved in cell division, survival, angiogenesis, and invasion in BC [15]. Therefore, receptor–protein associations are attracting increased interest concerning their role in hormone action as well as their potential use as therapeutic targets in hormonal diseases. Hormonal receptors are involved in BC progression at the hormone-dependent and hormone-refractory stages. Generating specific peptides derived from hormone receptor sequences can be useful tools to study the hormonal action responsible for cancer progression. Such peptides may also be developed as therapies that target steroid-receptor-expressing cancer cells [16].

BCER−, including triple-negative BC (TNBC), have few targeted treatment options; thus, they are mainly treated with chemotherapy. Unfortunately, chemotherapeutic efficacy is diminished by several drug resistance mechanisms that often occur in many cancer patients. Leptin signaling has been related to the proliferation and progression of both BCER+ and BCER− [17]. However, data available on the potential influence of leptin signaling on survival and response to chemotherapy of BC patients have been very limited. We hypothesize that leptin/OBR signaling decreases the survival of BCER− patients in part by increasing the expression of genes related to chemoresistance. Data from these studies will provide supporting evidence, indicating that targeting the leptin signaling pathway in highly aggressive obesity-related cancers may be an effective means to improving the success of BC treatment.

In this study, we have analyzed clinical, cellular, and molecular data obtained from The Cancer Genome Atlas (TCGA) database and TNBC cells treated with leptin, leptin signaling antagonists, and chemotherapeutics. This study provides evidence that high OBR mRNA expression and co-expression of leptin signaling targeted genes is associated with significantly decreased BC patient survival, particularly among those suffering from BCER−. This detrimental association was more evident in BC patients who received chemotherapy. Additionally, leptin signaling induced significant changes in the expression of genes involved in cancer progression and chemoresistance in TNBC cells. Overall, leptin-induced effects significantly impaired the cytotoxicity of chemotherapeutics on non-resistant and vinblastine-resistant TNBC cells.

## 2. Results

### 2.1. OBR and Leptin-Targeted Gene Co-Expression Correlates with Low Survival of BCER− Patients

**Figure 1:**
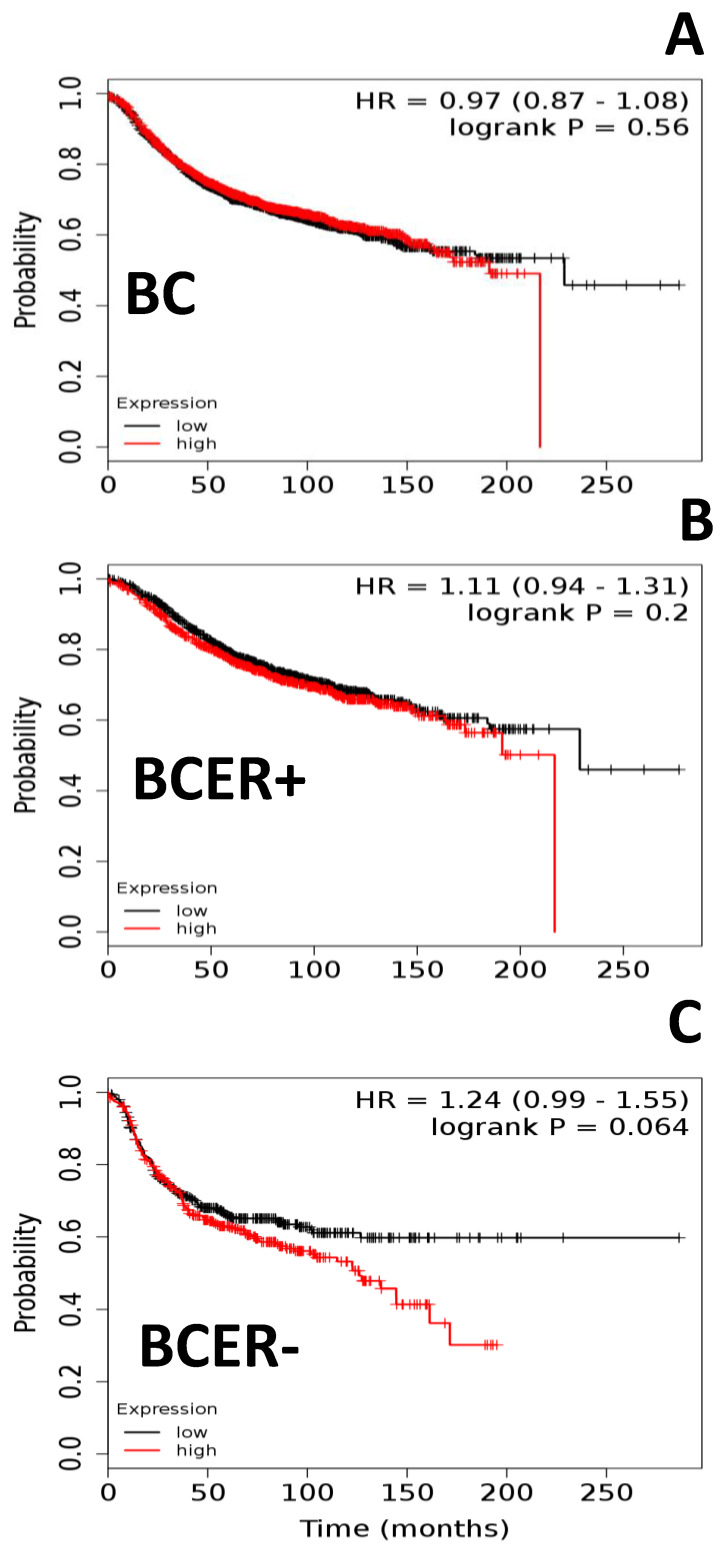
Leptin receptor (OBR) mRNA expression and survival of breast cancer (BC) patients. (**A**) Survival curves of patients (all breast cancer sub-types; *n* = 3951). (**B**) Survival curves of patients stratified by estrogen-receptor-positive BC (BCER+; *n* = 2061) and (**C**) estrogen-receptor-negative BC (BCER−; *n* = 801) subtypes. Kaplan–Meier survival plots were calculated for BC patients expressing low versus high levels of OBR mRNA according to data from the Cancer Genome Atlas (TCGA) [18,19,20,21]. Graphs depict relapse-free survival. Patients surviving beyond the timeline threshold (20 years and 10 months) were censored instead of excluded. Hazard ratio (HR) range and *p*-values (logrank P) were obtained for the BC subtypes using Kmplot.com software [18].

Gene expression was analyzed on TCGA datasets from BC samples to construct Kaplan–Meier survival curves and determine whether there is an association between high OBR expression and BC patient survival. TCGA mRNA analysis of BC tissue samples (all types; *n* = 3951) found no significant association between lower patient survival and high expression of OBR (Figure 1A).

To determine if the expression of ER could influence the association between OBR expression and survival, mRNA data from BCER+ (*n* = 2061) and BCER− (*n* = 801) tissues were analyzed. Results from BCER+ samples showed no association between high OBR expression and lower survival (Figure 1B). However, when similar analysis was done on BCER− patients, a marked trend (*p* = 0.06) was found, especially evident during the first 200 days after diagnosis, suggesting that high OBR expression is associated with lower survival (Figure 1C). Results from TNBC (*n* = 255) or basal BC (*n* = 186) samples did not show significant association between high OBR expression and lower survival (data not shown).

**Figure 2:**
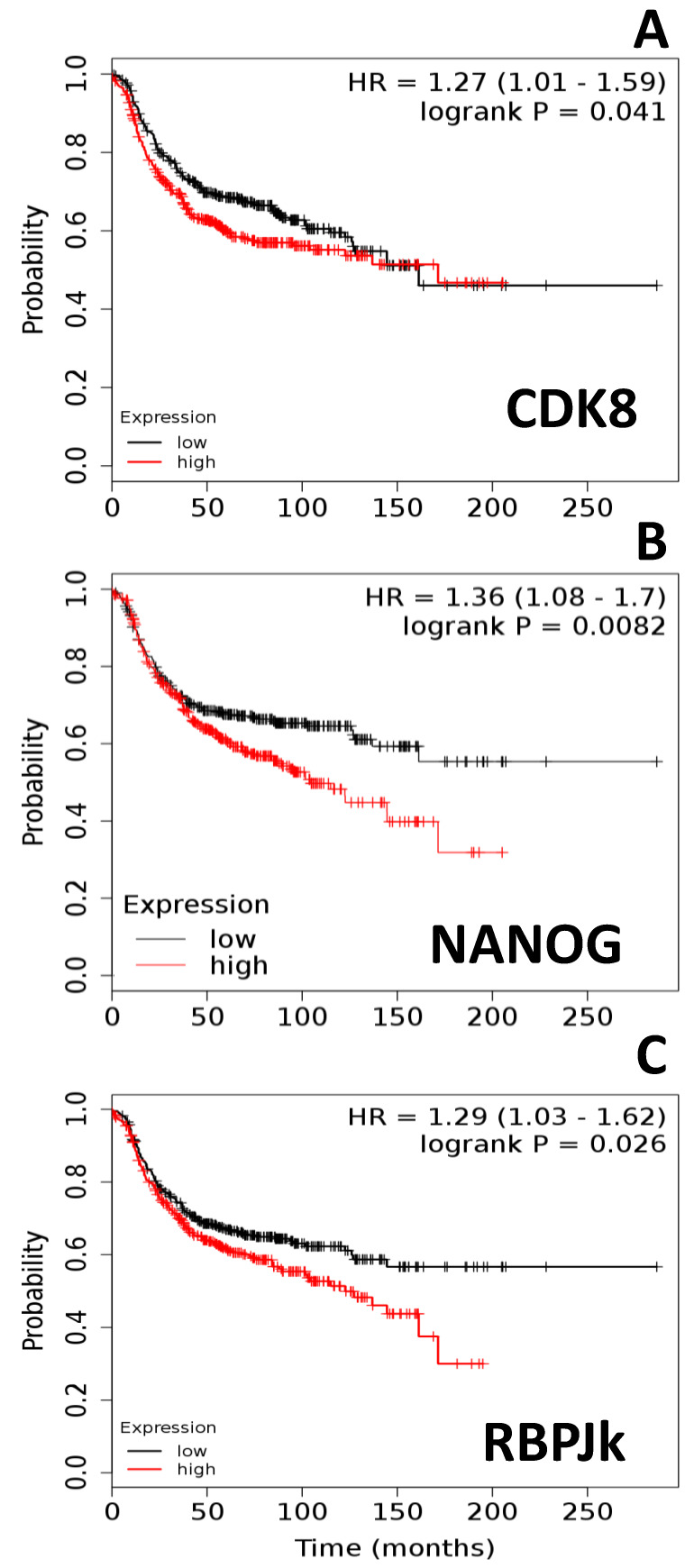
Expression of leptin-targeted gene mRNA reduces survival of ER-negative breast cancer patients (BCER−). Survival curves of patients (*n* = 801) with low and high expression of (**A**) CDK8, (**B**) NANOG, and (**C**) RBP-Jk. Kaplan–Meier survival plots were calculated using data from the Cancer Genome Atlas (TCGA) [18,19,20,21]. Graphs depict relapse-free survival. Patients surviving beyond the timeline threshold (20 years and 10 months) were censored instead of excluded. Hazard ratio (HR) range and *p-*values (logrank P) were obtained for the BCER− patients using Kmplot.com software [18].

Further, we asked if the expression of leptin signaling targeted genes (CDK8, NANOG, RBP-Jk) or their co-expression with OBR could be associated with lower BC patient survival. Interestingly, high expression of these leptin-targeted genes significantly decreased overall survival of BCER− patients. High expression of CDK8 in BCER− patients was significantly associated with reduced survival (*p* = 0.041) (Figure 2A). Moreover, high expression of NANOG (*p* = 0.0082; Figure 2B) or RBP-Jk (*p* = 0.026; Figure 2C) was also associated with poor overall survival outcomes in BCER− patients. This was not true for the high expression of CDK8 (*p* = 0.26) and RBP-Jk (*p* = 0.57) in BCER+ patients. However, the expression of the stem cell marker, NANOG, was associated with lower survival (*p* = 0.021) in BCER+ patients (BCER+ data not shown).

**Figure 3:**
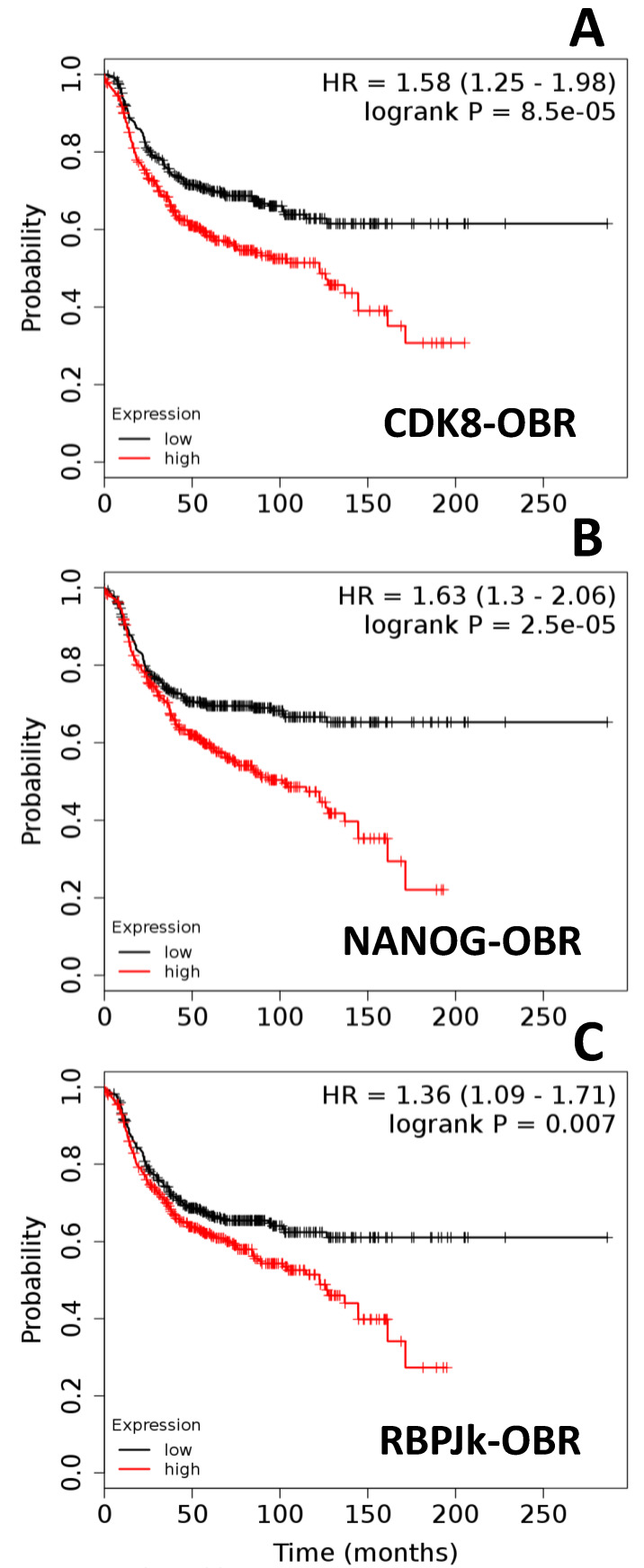
Co-expression of OBR and targeted genes decreases survival of ER-negative breast cancer patients (BCER−). Survival curves of patients (*n* = 801) with low and high expression of (**A**) CDK8-OBR, (**B**) NANOG-OBR, and (**C**) RBPJk-OBR. Kaplan–Meier survival plots were calculated using data from the Cancer Genome Atlas (TCGA) [18,19,20,21]. Graphs depict relapse-free survival. Patients surviving beyond the timeline threshold (20 years and 10 months) were censored instead of excluded. Hazard ratio (HR) range and *p-*values (logrank P) were obtained for the BCER− patients using Kmplot.com software [18].

Additional analyses showed that co-expression of OBR and CDK8 at high levels (*p* = 8.5 × 10^−5^; Figure 3A); OBR and NANOG (*p* = 2.5 × 10^−5^; Figure 3B), and OBR and RPB-Jk (*p* = 0.007; Figure 3C) in BCER− were significantly associated with lower patient survival. Similarly, high co-expression of OBR and CDK8 (*p* = 0.024) or of OBR and NANOG (*p* = 0.0008) also were associated with significantly decreased BCER+ patient survival. In contrast, high co-expression of OBR and RBP-Jk was associated with increased survival of BCER+ patients (*p* = 0.001) (data not shown).

The influence of the co-expression of OBR, CDK8, NANOG, and RBP-Jk was investigated in BCER− (*n* = 801) and BCER+ (*n* = 2062) patient datasets to examine further the relationships between high expression of OBR, leptin-targeted genes, and patient survival. A significant decrease in survival (*p* = 0.001) of BCER− patients was found when these genes were co-expressed (Figure S1A). However, the co-expression of the genes listed above was not associated (*p* = 0.13) with lower survival in BCER+ patients (Figure S1B).

**Figure 4:**
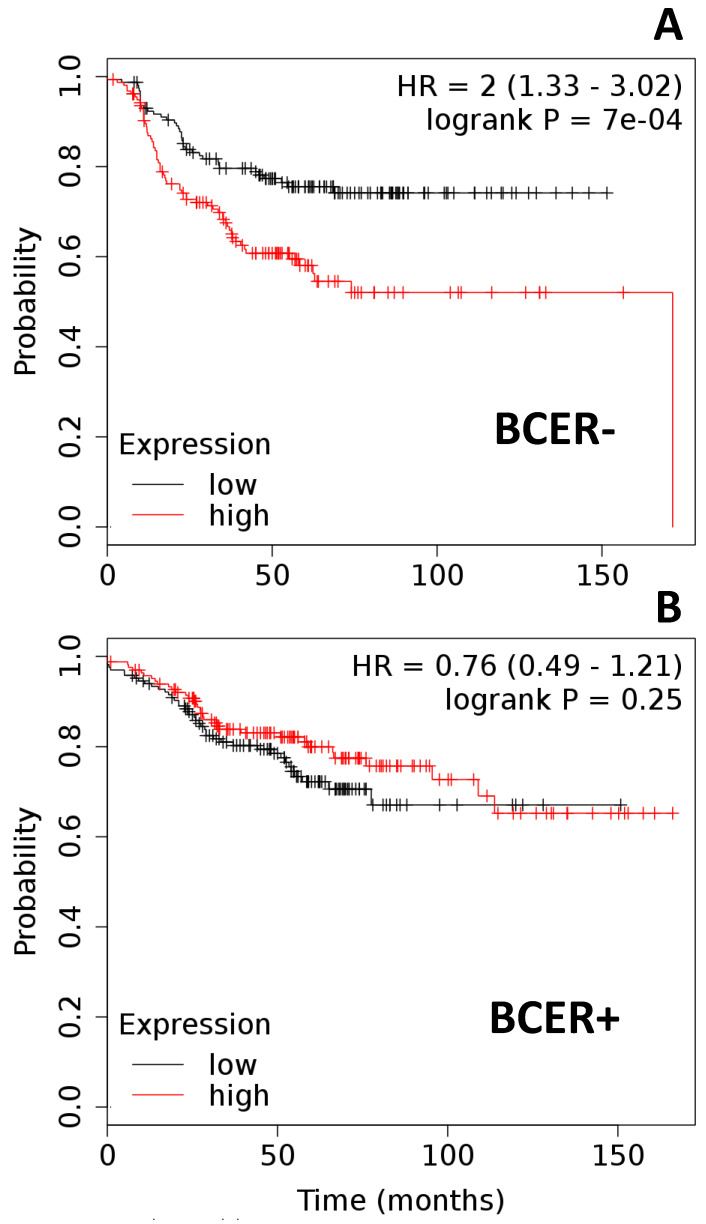
Co-expression of OBR and targeted genes and survival of ER-negative (BCER−) and -positive (BCER+) breast cancer patients treated with chemotherapeutics. (**A**) Survival curves of BCER− patients (*n* = 315) with low and high co-expression of CDK8–NANOG–RBPJk–OBR. (**B**) Survival curves of BCER+ patients (*n* = 331) with low and high co-expression of CDK8–NANOG–RBPJk–OBR. Kaplan–Meier survival plots were calculated using data from the Cancer Genome Atlas (TCGA) [18,19,20,21]. Graphs depict relapse-free survival. Patients surviving beyond the timeline threshold (20 years and 10 months) were censored instead of excluded. Hazard ratio (HR) range and *p-*values (logrank P) were obtained for the BC patients using Kmplot.com software [18].

We also investigated if the co-expression of OBR and leptin-targeted genes could influence the survival outcomes in BC patients treated with chemotherapy. The co-expression of OBR, CDK8, NANOG, and RBP-Jk (*p* = 7 × 10^−4^; Figure 4A) significantly impaired chemotherapy’s effects on BCER− patient survival (*n* = 315). Similar analyses in BCER+ patients (*n* = 331) showed that the co-expression of OBR and leptin-targeted genes CDK8, NANOG, and RBP-Jk (*p* = 0.25) did not affect patient survival (Figure 4B).

### 2.2. Leptin Impaired Chemotherapeutic Effects on TNBC Cells

To further investigate whether leptin could be a survival factor for BC cells treated with chemotherapeutics, TNBC cell lines that differentially respond to chemotherapeutics were treated with paclitaxel, cisplatin, leptin, and leptin signaling inhibitors (peptides and shRNA).

First, to assess the specificity of leptin effects, OBR expression in TNBC cells was determined. Additionally, leptin antagonists were used. Western blot analysis demonstrated that all cell lines used expressed the long and fully active OBR isoform (Figure S2A). Western blot analysis also showed decreased leptin-induced pSTAT3 levels in TBNC cells treated with antagonists of OBR (Figure S2B,C). In addition, OBR antagonists effectively inhibited the proliferative effects of leptin on TNBC cells (Figure S3A,B) and significantly reduced leptin-induced progression of the cell cycle (Figure S3C,D) but were not cytotoxic for non-malignant breast cells MCF 10A (Figure S4) or TNBC (MDA-MB468, MDA-MB231, and MDA-MB231 VBL-100; data not shown). Furthermore, the specificity of leptin effects was validated in MDA-MB231 cells where *OBR* expression was knocked down using shRNA. Optimal transduction was achieved with 10 µL of (1×) lentiviral preparation, which reduced OBR levels by 80%. This leads to a significant knockdown of OBR as compared to the parental (non-transduced) and vector control (Vec). There was a significant (50%) decrease of leptin-induced pSTAT3 in OBR shRNA knockdown cells. Indeed, leptin treatment in OBR shRNA knockdown cells did not induce changes in pSTAT3 or cell proliferation (Figure S5). This mirrored the results of blocking the leptin signaling pathway with C6 and mC6 leptin antagonists (see Figure S2).

**Figure 5:**
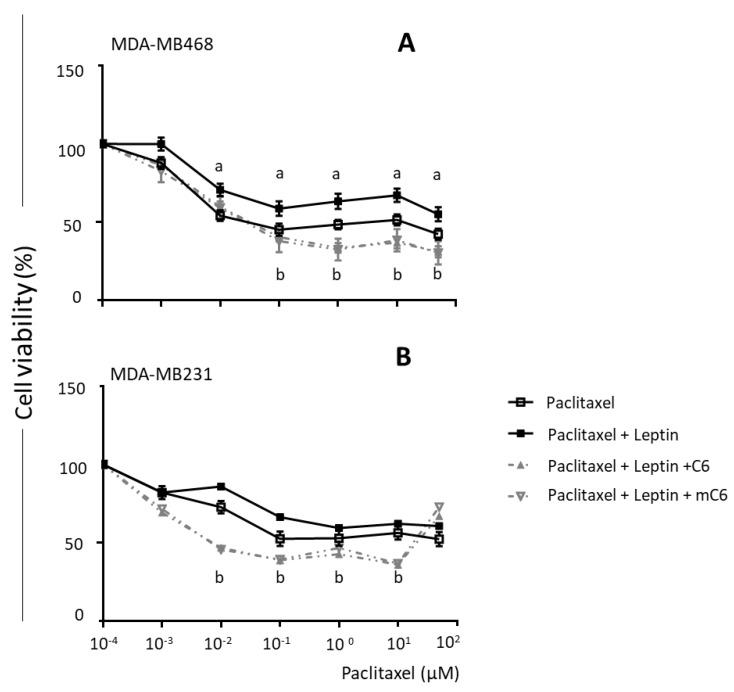
Leptin treatment increases survival of triple-negative breast cancer cells treated with paclitaxel. Paclitaxel dose–response effects on breast cancer cell survival: (**A**) MDA-MB468 and (**B**) MDA-MB231. Cells were treated with paclitaxel (P, 10^−4^–10^2^ μM); P + Leptin (L, 2.5 nM) and P + L + C6 and mC6 leptin antagonists (2.5 nM) for 72 h. CellTiter-Glo Assay was used to determine relative cell survival; “a”: *p* ≤ 0.05 compared to P; “b”: *p* ≤ 0.05 compared to P + L.

After assessing the specificity of leptin-induced changes in vitro, its effects on survival in TNBC cells treated with paclitaxel or cisplatin were investigated. As expected, TNBC cells (MDA-MB231 and MDA-MB468) that were sensitive to chemotherapeutics showed reduced survival when treated with paclitaxel and cisplatin (Figure 5). Leptin significantly decreased the toxicity of paclitaxel (0.01–50 µM) on MDA-MB468 cells (Figure 5A). Moreover, treatment of TNBC cells with leptin significantly increased the paclitaxel IC50 value (>0.01 µM). Similar effects were observed in MDA-MB231 cells treated with leptin and paclitaxel (Figure 5B). These leptin effects were reduced by the addition of OBR inhibitors C6 and mC6, which also increased the cytotoxic effects of paclitaxel in TNBC cells (Figure 5).

**Figure 6:**
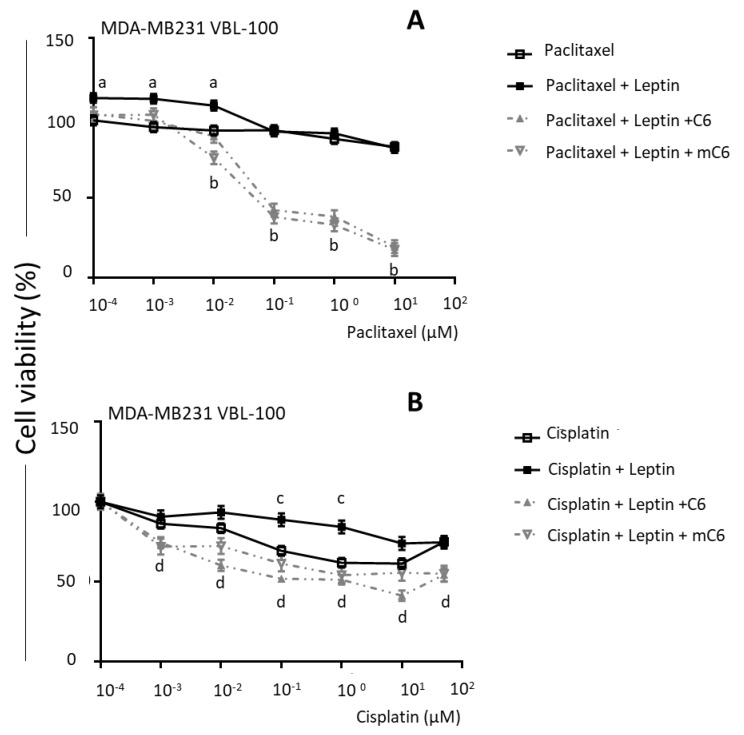
Leptin increases survival of chemoresistant triple-negative breast cancer cells treated with paclitaxel and cisplatin. (**A**) Paclitaxel and (**B**) cisplatin dose–response effects on survival of MDA-MB231 VBL-100 breast cancer cells. Cells were treated with paclitaxel (P, 10^−4^–10^2^ μM) or cisplatin (C, 10^−4^–10^2^ μM); P or C + Leptin (L, 2.5 nM) and P or C + L + C6 and mC6 leptin antagonists (2.5 nM) for 72 h. CellTiter-Glo Assay was used to determine relative cell survival; “a”: *p* ≤ 0.05 compared to P; “b”: *p* ≤ 0.05 compared to P + L; “c”: *p* ≤ 0.05 compared to C; “d”: *p* ≤ 0.05 compared to C + L.

In contrast, when vinblastine-resistant (MDA-MB231 VBL-100) cells were treated with paclitaxel (Figure 6A) or cisplatin (Figure 6B), the survival curves were unchanged compared to untreated cells. However, leptin increased the survival of MDA-MB231 VBL-100 treated with either paclitaxel or cisplatin. OBR inhibition re-sensitized TNBC chemoresistant cells to paclitaxel and cisplatin.

### 2.3. Leptin Increased the Expression of Key Proteins Associated with Survival and Motility in TNBC Cells Treated with Paclitaxel

**Figure 7:**
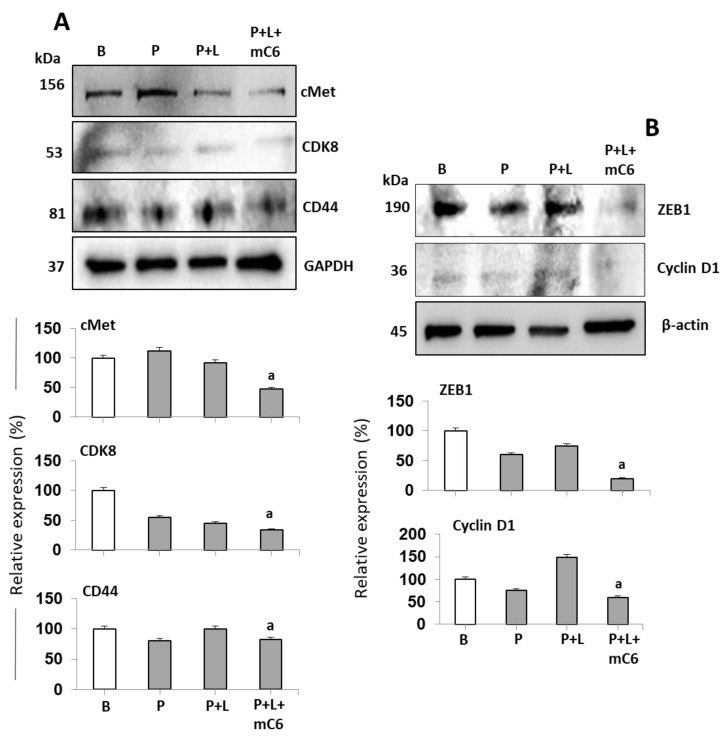
Inhibition of leptin signaling reduces the expression of key tumorigenic proteins in paclitaxel-treated triple-negative breast cancer cells. (**A**) Leptin antagonism effects on levels of cMet, CK8 and CD44 and (**B**) Cyclin D1 and ZEB1 in MDA-MB231 cells treated with paclitaxel. Cells were treated with paclitaxel (P, 0.5 mM); P + leptin (L, 6.25 nM) and P + L + mC6 leptin antagonist (12.5 nM) for 72 h. Western blot assays were used to determine protein levels. “B”: basal (no treatment). “a”: *p* ≤ 0.05 compared to P + L.

To further test whether leptin induces pro-survival effects of cells treated with chemotherapy, TNBC cells were incubated with paclitaxel and leptin for 72 h. As before, the specificity of leptin effects was assessed by the addition of mC6 (OBR inhibitor peptide). Inhibition of leptin signaling in paclitaxel-treated MDA-MB231 cells reduced the expression of proteins associated with cell cycle progression (Cyclin D1 and CDK8), survival (cMET and CD44), and epithelial to mesenchymal transition (ZEB1) (Figure 7).

However, analysis of the co-expression of OBR mRNA and additional leptin-targeted genes (MYC, CD44, and ZEB1) using TCGA breast cancer patient datasets did not show association with changes in survival either in BCER+ or BCER− patients. High co-expression of OBR and cMET showed significant association with lower survival in BCER− patients (*p* = 0.004; data not shown).

### 2.4. Leptin Induced the Expression of Proliferation and Chemoresistance-Related Genes in TNBC Cells

The effects of leptin on gene expression in TNBC cells were investigated using RNA-Seq. Differential gene expression (log FC changes ≥ 1.4) was used to identify significant changes in gene expression (*p* ≤ 0.05). These analyses resulted in statistically significant differentially expressed genes (DEG) in over 1000 genes when comparing control versus leptin-treated cells (data not shown).

**Figure 8:**
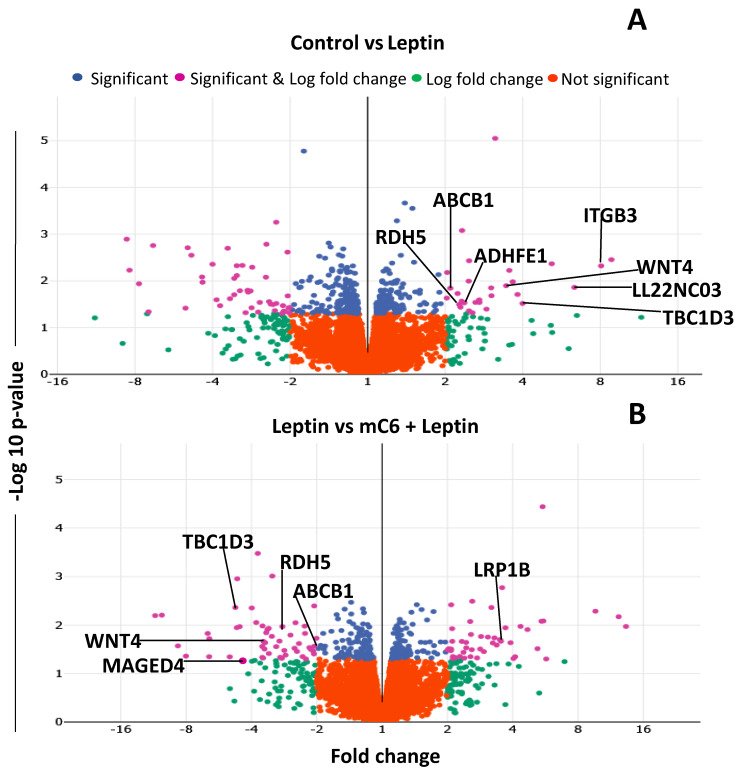
Volcano plots of leptin effects on changes in gene expression in triple-negative breast cancer cells. (**A**) Untreated cells (control) versus leptin-treated cells. (**B**) Leptin-treated cells versus leptin +mC6 treated cells. MDA-MB231 cells were untreated or treated with leptin (6.25 nM) or leptin plus mC6 leptin antagonist (5 nM) for 24 h. After RNA extraction and purification, RNA-Seq experiments were conducted using the Illumina NextSeq platform, and differentially expressed genes (DEGs) were determined by Log FC >1.4 and *p* ≤ 0.05. Positive and negative Log FC indicate upregulation and downregulation, respectively.

Volcano plots were created to determine changes in gene expression profiles of MDA-MB231 cells treated with leptin and leptin plus mC6 leptin antagonist (Figure 8). Results show that leptin signaling increases the expression of tumor progression and chemoresistance-related genes (ABCB1, WNT4, ADHFE1 [22], TBC1D3 [23], LL22NC03 [24], RDH5, ITGB3 [25]) in TNBC cells (Figure 8A). As expected, treatment with a leptin antagonist reversed several of these leptin-induced changes (Figure 8B). Moreover, leptin induces significant DEG of several genes associated with oncogenesis [23,26,27,28], proliferation [24,28], and metastasis [26,29,30,31] (Table 1).

**Figure 9:**
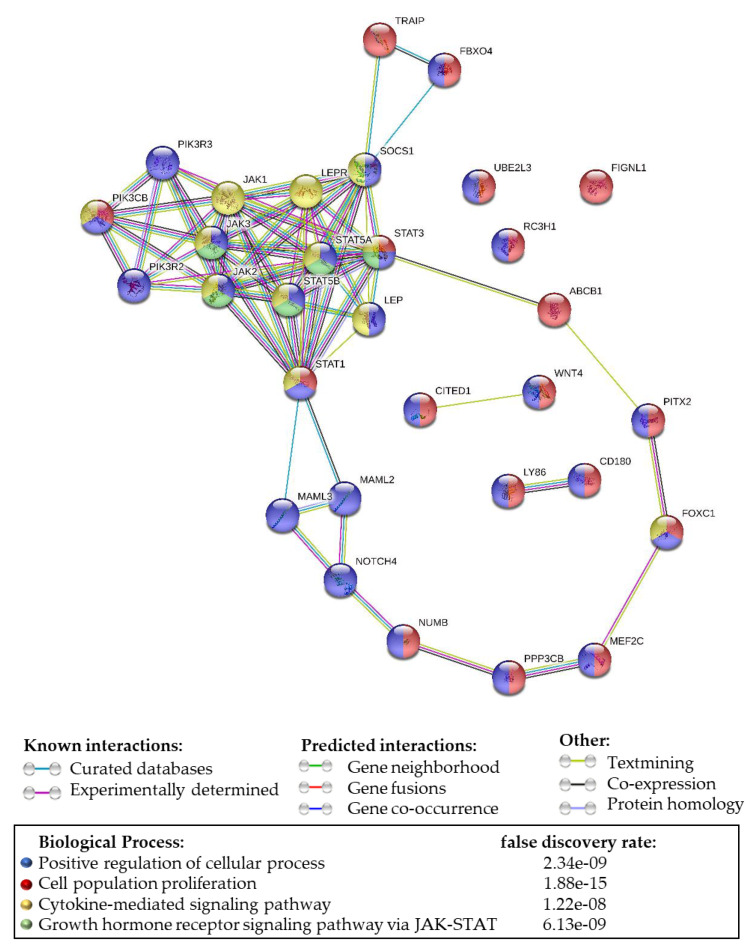
Leptin-induced protein–protein interaction (PPI) network in triple-negative breast cancer cells. PPI analysis of proteins from 18 genes (circles) that were up- or downregulated in MDA-MB231 cells following 24 h treatment with leptin (6.25 nM) created using STRING (online version 11.0) [40]. The lines connecting the proteins depict “known” or “predicted” interactions. Statistical analysis of the whole genome was calculated with STRING (online version 11.0) [40]. The PPI network enrichment *p*-value = 1.1 × 10^−12^, indicating that these interations within the network are significant. A total of 73 edges (protein–protein relationships) were discovered from 28 edges expected. Differentially expressed genes (DEGs) were determined by Log FC >1.4 and *p* ≤ 0.05.

Furthermore, whole-genome analyses of the protein–protein interaction network (String v.11.0 program [40]) for OBR and DEG leptin-induced proteins showed several predicted interactions (Figure 9). It is well known that the OBR signaling pathway mediates cell proliferation via the JAK-STAT pathway. As expected, this signaling pathway occupied a central position in the many interactions found. Canonical leptin signaling pathways include the activation of the PI3K cascade. Consequently, we found several protein–protein interactions involving PI3K isoforms. However, currently, there are no studies implicating leptin signaling as a modulator of drug resistance via the transporter functions of the ATP-binding transporter protein, ABCB1. Novel unpredicted interactions were found between leptin receptor, STAT3, and ABCB1, which has been linked to the development of drug resistance in cancer cells. Additionally, several new unexpected protein–protein interactions (edges) were identified in TNBC cells treated with leptin (see Figure 9 [40]; *p* = 1.1 × 10^−12^).

## 3. Discussion

Obesity as a cancer risk factor has been linked to excessive leptin levels in the bloodstream of overweight or obese individuals. From 1995 to 2014, obesity-related cancer incidences in the U.S were reported to have increased in people 24–49 years old in six different cancers [41]. It is also known that BC cells overexpress leptin and its receptor, OBR, which has been highlighted as a marker for the stimulation of cancer cell survival and progression [5,8,42]. Excess leptin can result in abnormal OBR signaling, leading to disease progression and poor prognosis in human obesity-related cancers [43]. Leptin canonical signaling pathways involve mainly the activation of the JAK2, PI3K, and MAPK signaling cascades. Present data from TNBC cells show several protein–protein interactions between OBR (LEPR, see Figure 9), JAK2/STAT, and PI3K. These signaling pathways were investigated previously for the development of targeted therapies for breast cancer [44,45]. In line with this, it is known that leptin binding to OBR increases S-phase cell cycle progression, angiogenesis, apoptosis evasion, invasion, and chemoresistance [46,47]. The results in our study demonstrate a correlation between expression of the leptin receptor (OBR) and leptin-induced genes in BCER− and provide data showing that signaling via the leptin receptor induces changes in genes associated with drug resistance in cancer.

Previously published data have established a relationship between leptin signaling and various growth-promoting pathways. Crosstalk between OBR and ER has been previously identified in BC, where the presence of ER and the activation of leptin signaling increased the proliferation and viability of BCER+ cells [48]. Leptin can transactivate ER and increase aromatase activity, which leads to the induction of estrogen synthesis [49,50]. Estrogen signaling can induce leptin and OBR expression reciprocally [51]. Moreover, it has been reported that estradiol administration increases leptin and OBR expression in ER-positive MCF-7 breast cancer cell line [50].

Leptin’s pleiotropic effects are linked to diverse processes that, if dysregulated, could contribute to the growth of cancer. We and others have shown that leptin signaling impacts several pathways involved in cancer, and specifically in BC development. Our more recent published data show that leptin is involved in a complex signaling network that integrates its developmental, pro-inflammatory, and pro-angiogenic effects, which are critical for leptin-induced cell proliferation, migration, angiogenesis, and self-renewal of BC stem cells [10]. In addition to its direct action through OBR and crosstalk with ER, leptin signaling can interact with growth factors, Notch, and inflammatory cytokine signaling to further affect BC risk, progression, recurrence, and mortality. Indeed, a complex crosstalk between Notch, IL-1, and leptin (NILCO) occurs in BC. NILCO could represent the integration of developmental, pro-inflammatory, and pro-angiogenic signals critical for leptin-induced cell proliferation/migration and angiogenesis in BC [11]. Leptin and NILCO signaling mediate the activation of cancer stem cells that can affect drug resistance [10]. Leptin seems to induce the expression of stem-cell self-renewal transcription factors NANOG, SOX2, and OCT4 in BC [52].

Recent data show that the adipokine leptin can modulate Notch/RBP-Jk signaling, thereby linking the obesity pandemic with cancer and chemoresistance [11,53]. RBP-Jk is a DNA-binding factor that mediates either Notch transcriptional repression or transcriptional activation. Activated Notch intracellular portion (NICD)-RBP-Jk complex displaces co-repressors at specific DNA binding sites that induce canonical signals increasing gene transcription (i.e., Hes, Hey, NFκB, cyclin D, c-MYC, etc.) [26]. Therefore, we have specifically investigated whether the co-expression of OBR and RBP-Jk (the Notch gatekeeper gene) and genes linked to Notch signaling (CDK8, NANOG) could affect BC patient survival.

In line with these reports, there was a clear difference in overall patient survival when they were stratified according to ER expression (i.e., BCER+ and BCER− subtypes). It seems that leptin signaling plays an important role leading to lower BCER− survival. Data analysis from TCGA mRNA gene chip expression in BCER− tissues showed that high mRNA expression of OBR, CDK8, NANOG, and RBP-Jk was associated with poorer survival of patients. In contrast, singular high expression of these genes was not associated with lower survival of BCER+ patients.

High co-expression of OBR, CDK8, NANOG, and RBP-Jk in BCER− patients was significantly associated with lower BCER− patient survival. This was also true for high co-expression of OBR and CDK8 or NANOG in BCER+ patients.

Furthermore, we show that the analysis of mRNA expression databases strongly suggests that high co-expression of OBR and leptin signaling target genes was associated with poor remission-free survival after chemotherapy and chemoresistance in BCER−. However, results did not show significant association between high OBR expression and lower survival after we investigated TNBC samples (from TCGA data sets). Potential factors that may contribute to this outcome could be the reduced number of samples and the menopausal and obesity status of patients, which were not described in the TCGA data set. It has been noted that data from BC studies among premenopausal obese women are still uncertain and somehow unreliable. Some reports showed that obese postmenopausal women are at a higher risk of developing BC. Other reports could find no association between serum levels of leptin in premenopausal or postmenopausal women and BC risk. Published data show a clear association between obesity and BC, although it seems to be restricted to ER- and progesterone-receptor-positive BC. Moreover, some studies have shown that ER-negative and TNBC tend to be inversely correlated with obesity after menopause [14].

Our results indicate that increased expression of leptin-signaling-induced genes may impair chemotherapeutic effects in BCER− patients. Thus, the co-expression of these genes may be used as clinical prognostic indicators for chemotherapy success in BCER− patients.

The implications of leptin signaling on chemoresistance in BC and other obesity-related cancers are not well understood [54,55]. We hypothesized that leptin antagonism may increase chemotherapy efficacy in BC [47,54]. Our data show that leptin is indeed an endogenous survival factor for BC. Remarkably, leptin signaling can induce resistance to chemotherapeutics in BCER− patients. Moreover, leptin treatment decreased the sensitivity of TNBC cells to paclitaxel and cisplatin. Interestingly, the use of novel antagonists, C6, and mC6, significantly increased the toxicity of paclitaxel and cisplatin in a chemoresistant-TNBC cell line (MDA-MB231 VBL-100). Treatment of TNBC cells with mC6 combined with paclitaxel also significantly decreased the levels of proteins associated with cell cycle progression (CDK8, Cyclin D1), epithelial to mesenchymal transition (ZEB1), and stemness (CD44, C-MET). Thus, inhibition of OBR function recovered the chemotherapeutic sensitivity of TNBC cells.

Despite the availability of clinical TCGA data, there is a dearth of data available that explores how leptin antagonism may influence gene expression changes in BC. Whole-genome sequencing was performed on TNBC cells treated with leptin. Data obtained show that leptin induced significant expression changes of genes associated with proliferation, cytokine signaling, and drug resistance of cancer cells [32,33,34,35,37,38,39].

The present studies show that the antagonism of leptin signaling reversed the gene effects of leptin in TNBC cells. Notably, inhibition of leptin signaling significantly reduced ABCB1 gene expression, which is well known to have high specificity for the elimination of paclitaxel from cancer cells [56]. Leptin also induced the expression of genes involved in BC progression (i.e., WNT4 [26], ADHFE1 [40], TBC1D3 [23], RDH5 [36], ITGB3 [25]). Moreover, analysis of the DEGs in TNBC cells stimulated with leptin showed an increased number of novel protein–protein interactions after the activation of OBR signaling.

Overall, the analysis of the present data from leptin signaling in TNBC cells and clinical survival data of BC patients and OBR/leptin-targeted gene expression indicates a translational potential for the use of inhibition of leptin signaling as adjuvant therapy. These data further support the notion that targeting the leptin signaling pathway could be an effective means to improve cancer treatment and reduce chemoresistance in BC.

## 4. Materials and Methods

### 4.1. Antibodies, Hormones, and Reagents

Human recombinant leptin was purchased from R & D Systems (Minneapolis, MN, USA). Monoclonal antibodies for phosphorylated STAT3 (pSTAT3), total STAT3 (tSTAT3), cyclin D1, c-MET, CD44, and ZEB1 were purchased from Cell Signaling Technologies (Danvers, MA, USA). CDK8 was purchased from Santa Cruz Biotechnology (Dallas, TX, USA). Additional monoclonal antibodies targeting α (91 kDa)/β (86 kDa) isoforms of pSTAT3 and tSTAT3 were purchased from Santa Cruz Biotechnology (Dallas, TX, USA). Leptin receptor (OBR) monoclonal antibody was purchased from Santa Cruz Biotechnology (Dallas, TX, USA). Paclitaxel and cisplatin were purchased from Sigma (St. Louis, MO, USA).

### 4.2. Leptin Peptide Antagonists

Design, synthesis, and purification of LPrA-2 were performed as described elsewhere [57]. Additional peptide antagonists C6, C8, C10, and mC6 (macrocyclic peptide antagonist) were designed, purified, and tested (pending patent) to determine whether leptin’s effects on TNBC cells were specific.

### 4.3. Cell Cultures

Non-malignant human mammary epithelial cells (MCF 10A) were obtained from ATCC (Manassas, VA, USA) and maintained in Mammary Epithelial Basal Medium (MEBM) supplemented with 0.001% human recombinant epidermal growth factor, hydrocortisone, bovine pituitary extract, 0.001% human recombinant insulin, 0.05% fetal bovine serum (FBS), and cholera toxin. Additionally, TNBC cell lines (MDA-MB231 and MDA-MB468) (obtained from ATTC) were maintained in Dulbecco’s Modified Eagle Medium (DMEM) supplemented with 10% FBS, 1% penicillin-streptomycin, 4.5 g/L d-Glucose, 1% l-Glutamine, and 110 mg/L sodium pyruvate. Cultures were maintained at 37 °C in 5% CO_2_ until the cellular monolayer was confluent. Cells were trypsinized using trypsin-EDTA and seeded in multi-well culture plates for cell cycle, MTT, and Western blot analyses. The human TNBC chemoresistant cell line, MDA-MB-231 VBL100 (VBL-100), was generated in the laboratory of Dr. Susan Bates (National Institute of Health, National Cancer Institute; Bethesda, MD, USA), as described in Huff et al. [58]. Cell cultures were routinely tested and found to be mycoplasma-free.

### 4.4. CellTiter-Glo Assay

MCF 10A cells were cultured as previously described and seeded in 96-well culture plates (5000 cells/well) and maintained until 70−80% confluent. Cells were then serum-starved for 24 h and treated with human recombinant leptin (1.25 nM) and leptin antagonists (1.25, 12.5, and 125 nM) for 24 or 48 h. CellTiter-Glo assay (Promega; Madison, WI, USA) was performed to determine cell viability.

### 4.5. Cell Cycle Progression Assay

Cancer cell lines were seeded (200,000 cells/well) in 6-well culture plates, maintained until 70–80% confluent, and serum-starved for 24 h. Cells were treated with leptin antagonists and human recombinant leptin (both at 1.25 nM) for 24 h (MDA-MB231) or 48 h (MDA-MB468). Cell cycle proliferation was analyzed using the Nexcellom Cellometer Vision CBA image cytometer (Nexcellom Bioscience, Lawrence, MA, USA).

### 4.6. MTT Assay

Cancer cell lines were seeded (5000 cells/well) in 96-well culture plates. The cells were treated as described for cell cycle progression assay. MTT assay was conducted to determine cell proliferation.

### 4.7. Western Blot

Protein concentration of cellular lysates was determined using the Bradford protein assay (Hercules, CA, USA) or Pierce BCA protein assay (Waltham, MA, USA). Cell lysates were combined (1:1) with Laemmli buffer (2×), and 30−50 µg of protein was loaded per lane on 4−15% (for pSTAT3 and tSTAT3) or Any kD gradient (for OBR) polyacrylamide gels. After electrophoresis, protein transfer was conducted using the Bio-Rad Trans-Blot Turbo system with 0.2 µm mini PVDF or nitrocellulose membranes as described elsewhere [11].

### 4.8. RNA Silencing of OBR in TNBC Cells

#### 4.8.1. Optimizing Leptin Receptor Knockdown in MDA-MB231 Cell Line

MDA-MB231 cells were seeded in 6-well plates (300,000 cells/well) and allowed to attach overnight. After aspirating media, 1 mL of Opti-MEM media was added per well. Lentiviral transduction particles (200 µL stock containing 1 × 10^6^ infectious units of virus; Santa Cruz) were used to transduce cells. Cells were transduced with vector control or lentiviral vector plasmid targeting human OBR (10 µL/mL (1×) or 20 µL/mL (2×)) for 48 h. Following transduction, cells were grown in complete culture media supplemented with puromycin (2 µg/mL). Cells were lysed, and Western blot analysis for OBR expression was performed.

#### 4.8.2. Detection of Leptin Signaling in OBR shRNA Knockdown Cells

MDA-MB231 parent/wild type, shRNA vector control, or OBR shRNA knockdown (1X transduced) cells were seeded in 6-well plates (200,000 cells/well). Following 24 h serum starvation, cells transduced with shRNA vector control or OBR shRNA were incubated with human recombinant leptin (2.5 nM) for five minutes. Western blot analysis was performed with 30–50 µg of protein/lane on 4%–15% polyacrylamide gels. After protein transfer, nitrocellulose membranes were probed with pSTAT3 and tSTAT3 monoclonal antibodies.

### 4.9. Combined Leptin Peptide Antagonist-Chemotherapy Treatment

TNBC cell lines (MDA-MB231, MDA-MB468, and VBL-100) were seeded in 96-well culture plates (5000 cells/well) and maintained until 70%–80% confluent. Cells were then serum-starved for 24 h and treated with increasing concentrations of paclitaxel or cisplatin for 72 h in combination with C6 or mC6 in the presence of leptin. MDA-MB231 and MDA-MB468 cells treatments: paclitaxel or cisplatin (0.0001–50 µM) in the presence of leptin antagonist and leptin (both at 2.5 nM); paclitaxel or cisplatin alone; chemotherapeutic combined with leptin. VBL-100 cell line was treated similarly with paclitaxel or cisplatin (0.0001–10 µM) plus leptin and antagonist, as described above.

### 4.10. RNA Extraction and RNA-Seq

MDA-MB-231 cells were plated in 6-well plates (200,000 cells/well) and serum-starved for 18–24 h. Then, cells were treated with leptin antagonists and human recombinant leptin (6.25 nM) for 24 h. Immediately following treatment, the cells were lysed at room temperature using Buffer RLT Plus supplemented with β-mercaptoethanol. Residual DNA removal and total RNA extraction were performed using the RNeasy Plus Mini Kit (Qiagen). RNA concentration and integrity number (RIN) score were determined. Samples with RIN scores ≥ 9.8 on a scale of 1–10 were used to prepare libraries from total RNA using the SureSelect Strand-Specific RNA Library Prep kit. Next, mRNA libraries were sequenced on the NextSeq platform with 75 × 75 bp paired-end reads. Sequencing data were analyzed using the NIH CBIT-CCBR Pipeliner. Raw paired-end data files (.fastq) were uploaded to the CCBR RNA-Seq Pipeliner, and initial quality control of raw data was determined. Adapter clipping (read trimming) was performed using Trimmomatic. STAR two-pass alignment was used to align trimmed reads to human genome 19 (hg19). FeatureCounts was used to quantify, and TMM was performed for normalization. Differentially expressed genes (DEG) were determined between samples using EdgeR platform. Significant DEG was defined for log2 fold (Log FC) changes ≥ 1.4 and *p* ≤ 0.05.

### 4.11. Breast Cancer Patient Kaplan–Meier Survival Curves

Kaplan–Meier (KM) plotter (http://kmplot.com) was used to create curves showing overall survival [18]. mRNA gene expression of clinical BC patient datasets from Gene Expression Omnibus (GEO) and TCGA were interrogated for this study [19]. High and low mRNA expression of genes associated with OBR signaling were depicted in KM survival curves. Hazard ratios and *p*-values were calculated by the KM plotter program; *p*-value ≤ 0.05 was considered statistically significant.

### 4.12. Protein-Protein Interaction (PPI) Analysis of Differentially Expressed Genes (DEG)

After performing RNA-Seq of leptin-treated MDA-MB231 cells (RNA-Seq), the STRING program (online v.11) was used to created a network of PPI. A total of 18 up-or-downregulated genes were entered into the STRING program. Novel interactions between the 18 proteins derived from DEG and several predicted interactions within this network were found to be significant (*p*-value = 1.1 × 10^−12^) [40].

### 4.13. Statistical Analysis

One-way ANOVA followed by multiple comparison tests were performed to determine statistical significance between groups using GraphPad Prism version for Windows (GraphPad Software, La Jolla, CA, USA, www.graphpad.com). Data are expressed as a mean of a minimum of three different experiments ± SEM. A *p*-value ≤ 0.05 was considered statistically significant. Statistical analysis of the PPI network was calculated within the STRING program (online v.11) [40]. The STRING program intergrates and ranks the protein interactions by comparing five main data sources: 1. Genomic context predictions; 2. High-throughput lab experiments; 3. Conserved co-expression; 4. Automated textmining and; 5. Previous knowledge in databases. Consequently, STRING used the five main data sources to create false discovery rates and *p*-value.

## Figures and Tables

**Table 1:** Leptin-induced differential expression of genes associated with cancer progression in triple-negative breast cancer cell line.

Gene	Control vs. Leptin		Leptin vs. Leptin + mC6		Function in Cancer
	*Log FC*	*p-Value*	*Log FC*	*p-Value*	
***WNT4***	3.45	0.00	−2.88	0.03	Oncogenesis, regulation of cell fate and development [26]
***ADHFE1***	2.37	0.03	−2.34	0.04	Oncogenesis and metabolic reprograming [22]
***TBC1D3***	3.99	0.03	−4.66	0.04	Oncogenesis [23]
***ABCB1***	2.09	0.01	−1.56	0.13	Multidrug resistance [29]
***LL22NC03***	6.35	0.01	−1.47	0.08	Proliferation [24]
***MAGED4***	5.20	0.13	−8.13	0.05	Lymph node BC metastasis [30]
***SOST***	−1.39	0.37	3.57	0.01	Inhibition of WNT pathway [32]
***LRP1B***	−1.56	0.37	3.49	0.01	Tumor suppressor activity in non-small cell lung cancer [33]
***EIF4BP7***	−1.13	0.75	2.27	0.02	Proliferation and overexpression of oncogenes [28]
***RARB***	−1.48	0.26	2.25	0.04	Tumor suppressors [34]
***DDK3***	1.48	0.19	−2.11	0.02	Cancer cell growth, metastasis and chemoresistance [35]
***RDH5***	2.27	0.03	1.01	0.99	Tumorigenesis [36]
***NEED8***	−1.09	0.11	−3.78	0.11	Cancer cell growth, survival and activation of ubiquitin ligase complexes [37]
***TMOD1***	−	−	−3.95	0.03	Lymph node metastasis in oral cancer [31]
***FOXD4L1***	−	−	−4.53	0.03	Cell cycle regulation [38]
***TAP2***	−1.01	0.75	−5.79	0.01	Multidrug resistance (ABC protein transporter) [39]
***ITGB3***	8.06	0.00	1.01	0.87	Cell migration and invasion [25]

## Supplementary Materials

Supplementary materials can be found at https://www.mdpi.com/1422-0067/21/11/3794/s1.
