# Supplementary material for: Leptin Signaling Affects Survival and Chemoresistance of Estrogen Receptor Negative Breast Cancer
Source: Int J Mol Sci. 2020 May 27;21(11):3794. doi: 10.3390/ijms21113794 (PMC7311967; doi:10.3390/ijms21113794)
Supplement: Supplementary file 1 [file ijms-21-03794-s001.zip › Fig 1_Suppl.pdf]

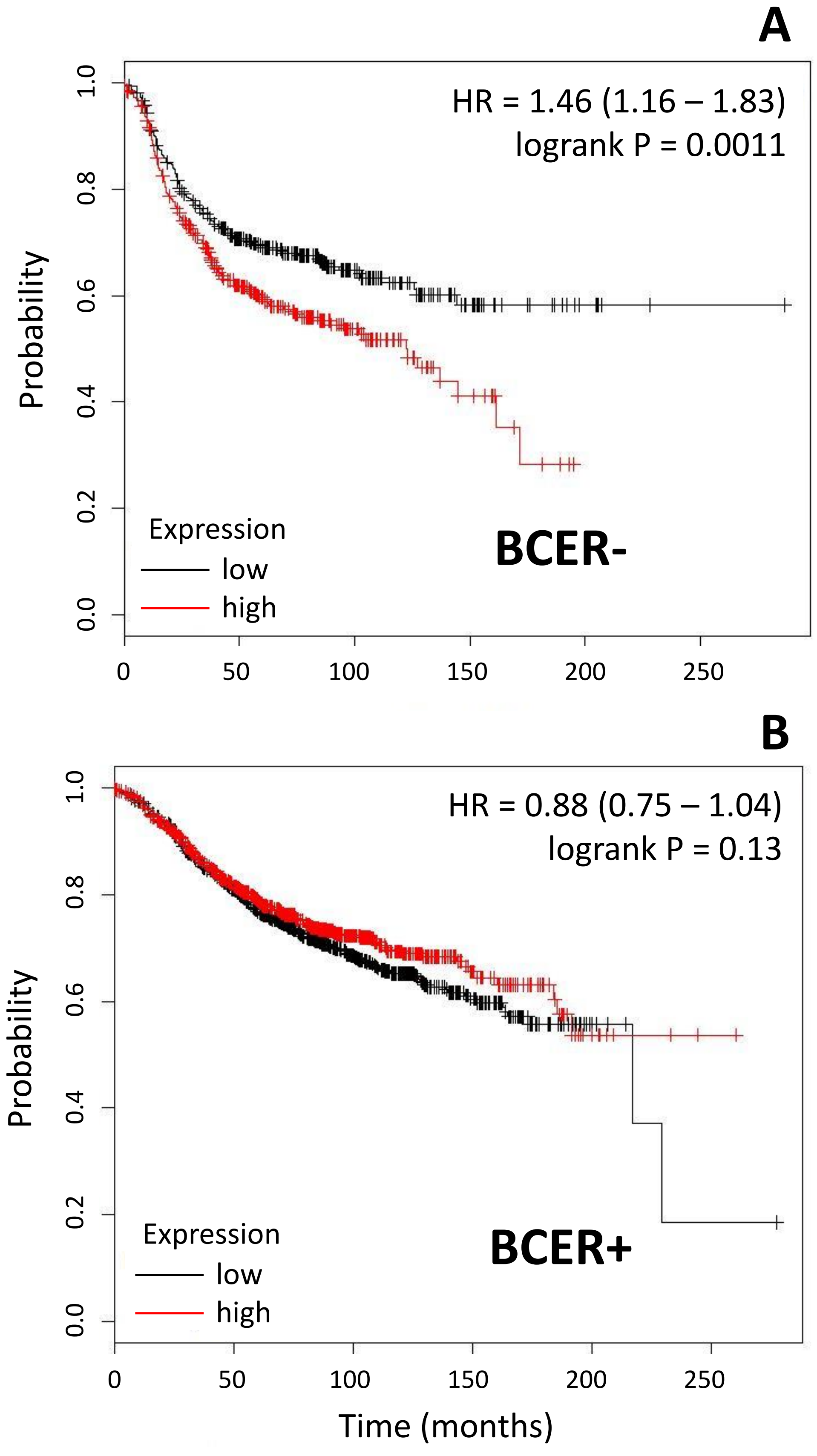

Figure S1. Survival of ER negative (BCER-) and positive (BCER+) breast cancer patients with co-expression of OBR and targeted genes. (A) Survival curves of BCER- patients (n=801) with low and high co-expression of CDK8-NANOG-RBPJk-OBR. (B) Survival curves of BCER+ patients (n=2061) with low and high co-expression of CDK8-NANOG-RBPJk-OBR. Kaplan-Meier survival plots were calculated using data from the Cancer Genome Atlas (TCGA). Graphs depict relapse free survival. Patients surviving beyond the timeline threshold (20 years and 10 months) were censored instead of excluded. Hazard ratio (HR) range and P values (logrank P) were obtained for the BC patients using Kmplot.com software [46].
