# Supplementary material for: Leptin Signaling Affects Survival and Chemoresistance of Estrogen Receptor Negative Breast Cancer
Source: Int J Mol Sci. 2020 May 27;21(11):3794. doi: 10.3390/ijms21113794 (PMC7311967; doi:10.3390/ijms21113794)
Supplement: Supplementary file 1 [file ijms-21-03794-s001.zip › Fig 2_Suppl.pdf]

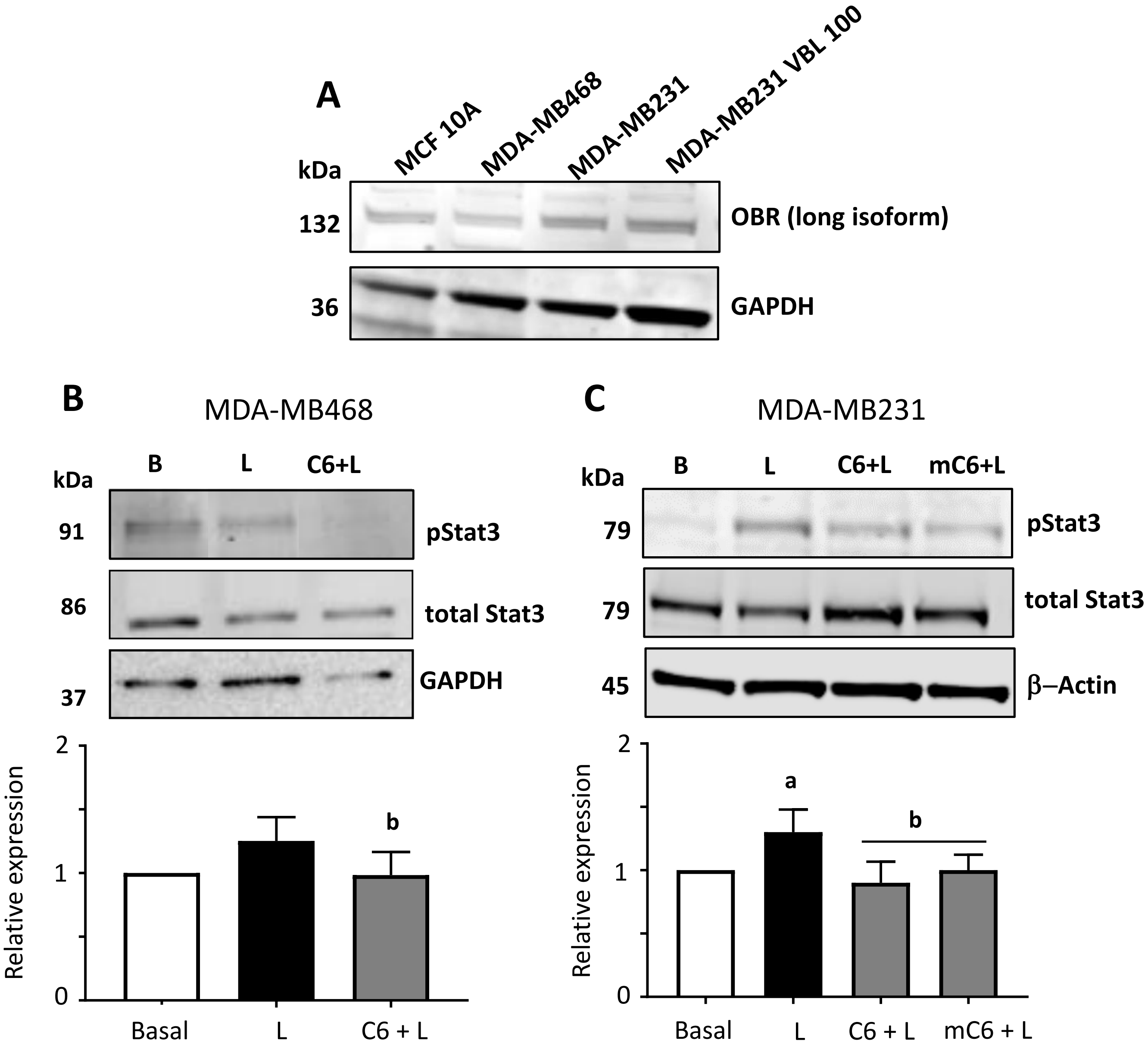

Figure S2. OBR expression and leptin signaling antagonism in triple negative breast cancer cells. (A) Expression of OBR long isoform was detected by Western blot (WB) in all breast cells used: non-malignant breast cells (MCF 10A), and triple negative breast cancer cell lines (TNBC: MDA-MB468, MDA-MB231, and vinblastine resistant cells MDA-MB231 VBL). Leptin induced phosphorylation of Stat3 (pStat3) was determined via WB in TNBC. The addition of leptin antagonists (C6 and mC6) abrogated leptin induced pStat3 in (B) MDA-MB468 and (C) MDA-MB231 cells. Graphs represent quantitative analysis of pSTAT3. All data were derived from a minimum of three independent experiments using different cell preparations. “a”: $p \leq 0.05$ compared to Basal and “b”: $p \leq 0.05$ compared to leptin.
