# Supplementary material for: Leptin Signaling Affects Survival and Chemoresistance of Estrogen Receptor Negative Breast Cancer
Source: Int J Mol Sci. 2020 May 27;21(11):3794. doi: 10.3390/ijms21113794 (PMC7311967; doi:10.3390/ijms21113794)
Supplement: Supplementary file 1 [file ijms-21-03794-s001.zip › Fig 3_Suppl.pdf]

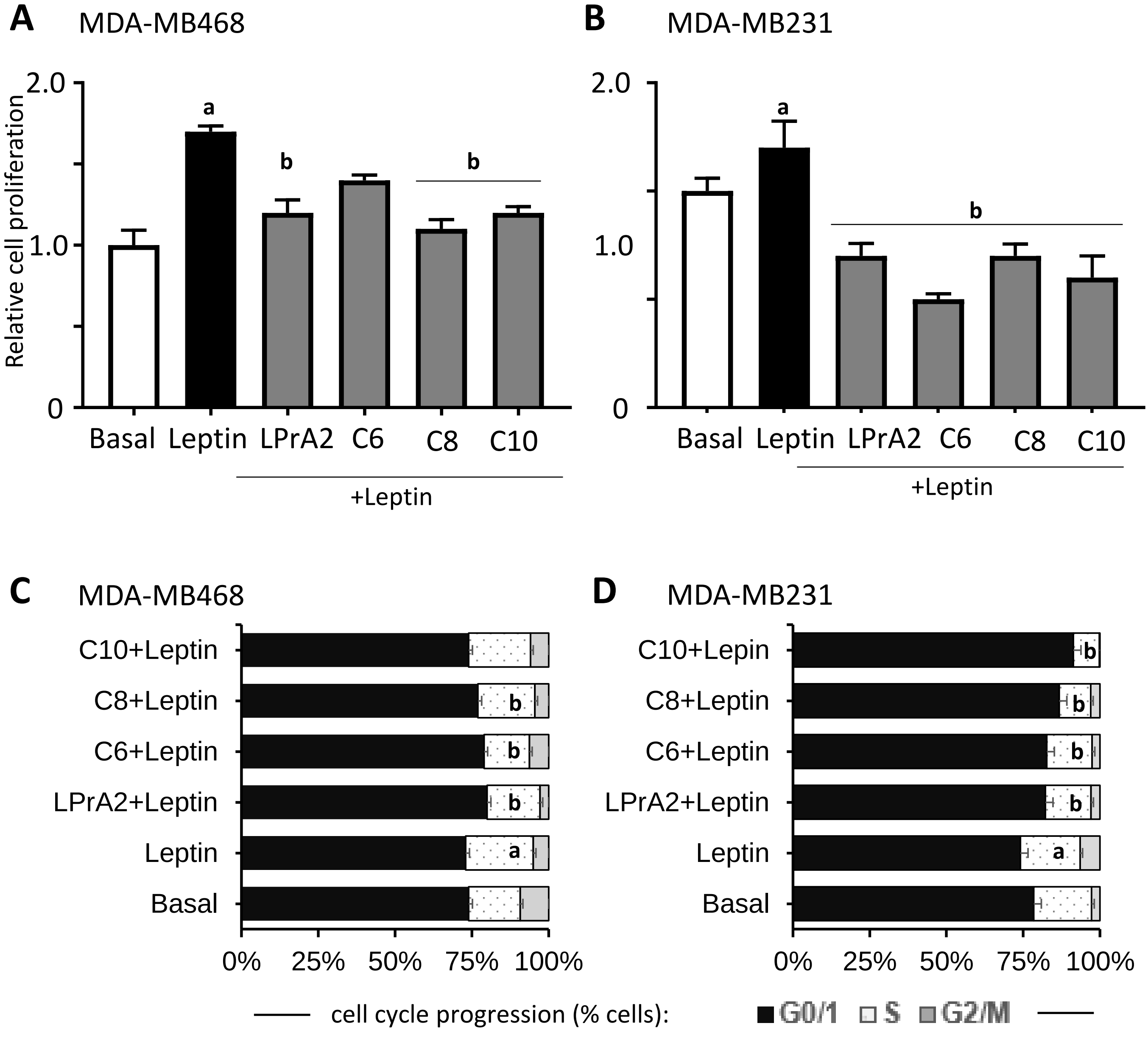

Figure S3. Leptin-induced proliferation and cell cycle progression of triple negative breast cancer cells are effectively inhibited by peptide antagonists. Leptin-induced cell proliferation of (A) MDA-MB468 and (B) MDA-MB231 triple negative breast cells (TNBC) was inhibited by peptide antagonists (LPrA2, C6, C8 and C10). Leptin-induced S-phase progression of (C) MDA-MB468 and (D) MDA-MB231 cells was inhibited by peptide antagonists. TNBC (5x103 cells/well; 96-well plates) were grown until 70-80% confluent, and serum starved for 18-24hrs. Then, TNBC were treated with leptin and leptin antagonists for 24hr. MTT assay was conducted to determine cell proliferation. Cell cycle progression was determined using a Cellometer (Vision CBA image cytometer; Nexcellom Bioscience). Leptin and peptides antagonists (LPrA2, C6, C8, C10; 1.25nM). All data were derived from a minimum of three independent experiments using different cell preparations. “a”: p ≤0.05 compared to Basal and “b”: p≤0.05 compared to leptin.
