# Supplementary material for: Leptin Signaling Affects Survival and Chemoresistance of Estrogen Receptor Negative Breast Cancer
Source: Int J Mol Sci. 2020 May 27;21(11):3794. doi: 10.3390/ijms21113794 (PMC7311967; doi:10.3390/ijms21113794)
Supplement: Supplementary file 1 [file ijms-21-03794-s001.zip › Fig 4_Suppl.pdf]

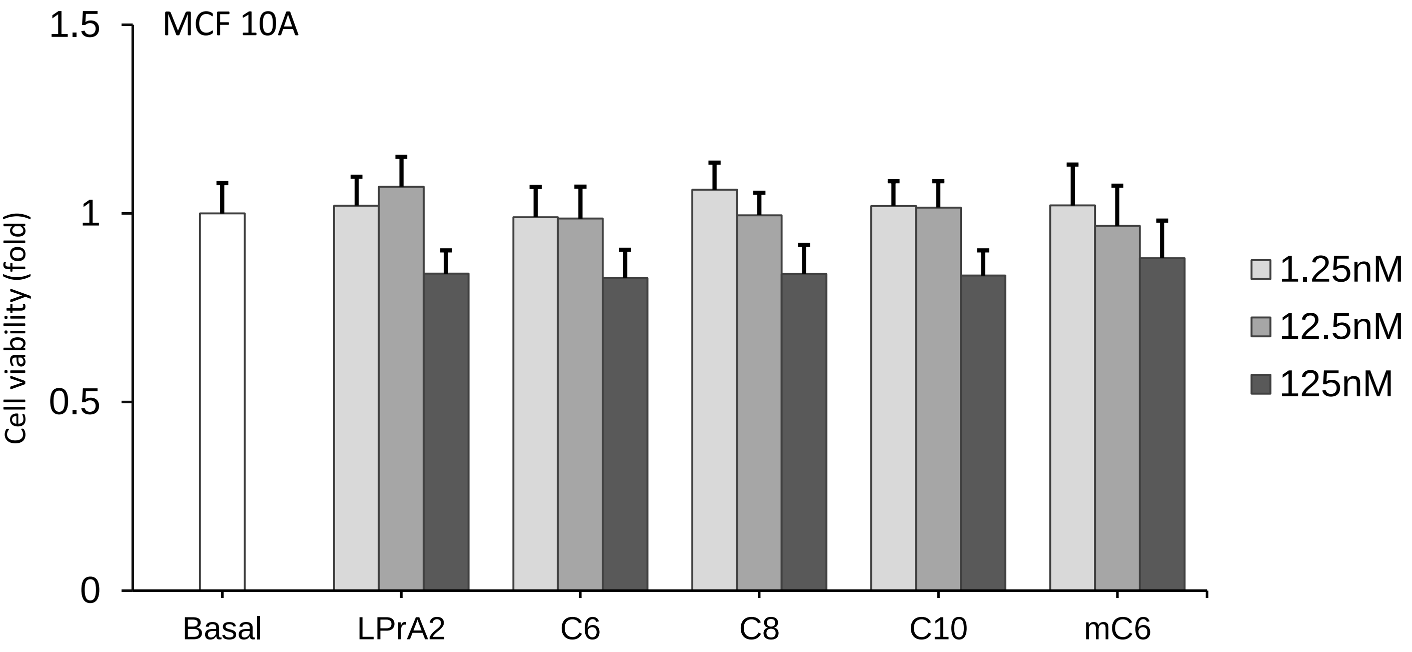

Figure S4. Leptin peptide antagonists are non-toxic for breast cells. MCF 10A cells (5x103 cells/well;96-well plates) were cultures until 70-80% confluent. Then, cells were serum starved for 18-24hrs and treated with leptin antagonists (LPrA2, C6, C8, C10, mC6; 1.25, 12.5,125nM) for 48hrs. Cell viability was determined by measuring ATP concentration in solution using either MTT assay or Cell-titer Glo assay. All data were derived from a minimum of three independent experiments using different cell preparations.
