# Supplementary material for: Leptin Signaling Affects Survival and Chemoresistance of Estrogen Receptor Negative Breast Cancer
Source: Int J Mol Sci. 2020 May 27;21(11):3794. doi: 10.3390/ijms21113794 (PMC7311967; doi:10.3390/ijms21113794)
Supplement: Supplementary file 1 [file ijms-21-03794-s001.zip › Fig 5_Suppl.pdf]

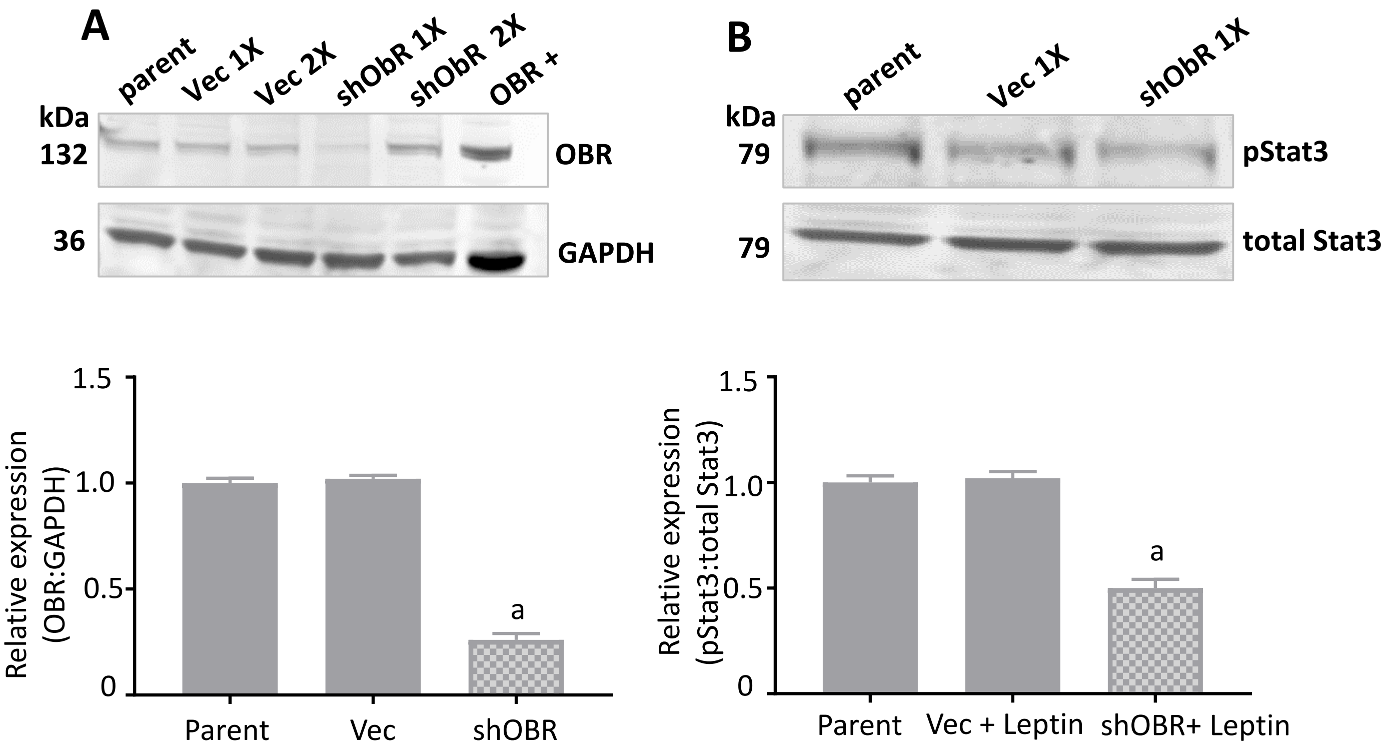


Figure S5. Knockdown of OBR by shRNA in triple negative breast cancer cells. (A) MDA-MB231 cells were transfected with shRNA vector control (Vec) and OBR shRNA [both at 10 µl (1X) and 20 µl (2X)]. The transfected cells were cultured (200,000 cells/well; 6-well plates) for 24 hrs. Western blot analysis (WB) of OBR expression in MDA-MB231 cells transfected with OBR shRNA or vector control (OBR+: positive control for OBR antibody). (B)WB analysis of leptin-induced pStat3 in knockdown and control MDA-MB231 cells. After 24hr serum starvation, transfected cells were incubated with leptin (2.5nM) for 5 min. WB was performed with 30 to 50µg of protein/lane on 4-15% polyacrylamide gels. Graphs represent quantitative analysis of OBR and pSTAT3. All data were derived from a minimum of three independent experiments using different cell preparations. “a”: p ≤0.05 compared to Vec + leptin.
